# Multi-Scale Aggregation Graph Neural Networks Based on Feature Similarity for Semi-Supervised Learning

**DOI:** 10.3390/e23040403

**Published:** 2021-03-28

**Authors:** Xun Zhang, Lanyan Yang, Bin Zhang, Ying Liu, Dong Jiang, Xiaohai Qin, Mengmeng Hao

**Affiliations:** 1 Beijing Key Laboratory of Big Data Technology for Food Safety, School of Computer Science and Engineering, Beijing Technology and Business University, Beijing 100048, China; zhangxun@btbu.edu.cn (X.Z.); yly_951010@yeah.net (L.Y.); zb18041963006@163.com (B.Z.); qxh_4929@163.com (X.Q.); 2 Key Laboratory of Resources Utilization and Environmental Remediation, Institute of Geographic Sciences and Natural Resources Research, Chinese Academy of Sciences, Beijing 100101, China; haomm.16b@igsnrr.ac.cn

**Keywords:** graph analysis, graph neural network, semi-supervised learning, neighborhood aggregation

## Abstract

The problem of extracting meaningful data through graph analysis spans a range of different fields, such as social networks, knowledge graphs, citation networks, the World Wide Web, and so on. As increasingly structured data become available, the importance of being able to effectively mine and learn from such data continues to grow. In this paper, we propose the multi-scale aggregation graph neural network based on feature similarity (MAGN), a novel graph neural network defined in the vertex domain. Our model provides a simple and general semi-supervised learning method for graph-structured data, in which only a very small part of the data is labeled as the training set. We first construct a similarity matrix by calculating the similarity of original features between all adjacent node pairs, and then generate a set of feature extractors utilizing the similarity matrix to perform multi-scale feature propagation on graphs. The output of multi-scale feature propagation is finally aggregated by using the mean-pooling operation. Our method aims to improve the model representation ability via multi-scale neighborhood aggregation based on feature similarity. Extensive experimental evaluation on various open benchmarks shows the competitive performance of our method compared to a variety of popular architectures.

## 1. Introduction

Convolutional neural networks (CNNs) [1] demonstrate state-of-the-art performance in a variety of learning tasks for processing 1D, 2D, and 3D Euclidean data, such as videos, acoustic signals, and images. However, the convolution operation is not applicable to deal with non-Euclidean data such as graphs, since each node may have a different number of adjacent nodes, and it is difficult to perform convolution operations using a convolution kernel of the same size.

In recent years, an increasing number of applications have represented data in the form of graphs. For example, in e-commerce, graph-based learning systems can leverage the interaction between users and products to make highly accurate recommendations. In chemistry, molecules are modeled as graphs that require the identification of their biological activity for drug discovery. In citation networks, papers are linked through citations, so they need to be grouped. Graph deep learning models have achieved great success in modeling relational data, including link prediction [2,3,4] graph classification [5,6], and semi-supervised node classification [7,8].

There are many approaches for leveraging deep learning algorithms on graphs. Node embedding methods use random walks or matrix factorization to directly train an individual node embedding, often without using node features and usually in an unsupervised manner, such as DeepWalk [9], LINE [10], and node2vec [11]. However, these are unsupervised algorithms that ignore the feature attributes of nodes. Therefore, they cannot perform node classification tasks in an end-to-end manner. Unlike previous methods based on random walking, the use of neural networks in graphs has been extensively studied in recent years. Examples for these include ChebNet [12], MoNet [13], GCN [8], and SSE [14], SGC [15]. Among these categories, this class of message-passing algorithms has received special attention because of its flexibility and good performance.

Recently, there has been increasing research interest in applying convolutional operations on graphs. These graph convolutional networks (GCNs) [8,15] are based on the neighborhood aggregation scheme that combines information from neighborhoods to generate node embedding. Compared with traditional methods, GCNs achieve promising performance in various tasks (e.g., node classification [8,16] and graph classification [12]). Nevertheless, GCN-based models are usually shallow, which limits the scale of the receptive field. When GCN is set up as a two-layer network, there is usually a better classification effect. However, the two-layer GCN model only aggregates information from 1-hop and 2-hop neighbors for each node; due to the limitation of the receptive field, it is difficult for this model to obtain sufficient global information. However, simply adding more layers to the GCN model will degrade the classification performance. According to the explanation in [17], each GCN layer essentially acts as a form of Laplacian smoothing, and as the number of layers increases, the hidden layer representations of all nodes will tend to converge to the same value, which will lead to over-smoothing [17,18]. Although some methods [19,20] attempt to obtain more global information through deeper models, they are either unsupervised models or require many training examples. Consequently, they still cannot solve the semi-supervised node classification task well.

Furthermore, for the semi-supervised learning, GCN-based models use a symmetric normalized adjacency matrix as the aggregation matrix to aggregate local information. However, the normalized adjacency matrix can only simply aggregate feature information from neighboring nodes for the target node. The original feature distribution relationship between the target node and its neighboring nodes is not considered in the process of aggregating information, which will result in failure to distinguish the relative importance of neighboring nodes for the target node. These neighboring nodes whose original feature distribution is closer to the target node should have a larger aggregation weight when aggregating information.

To solve the above-mentioned issues, in this paper, we propose a multi-scale aggregation graph neural network based on feature similarity (MAGN). We first construct a similarity matrix as the aggregation matrix by calculating the original feature similarity between adjacent node pairs. The similarity matrix is used as the aggregation matrix, which can distinguish the relative importance of neighboring nodes for the target node according to the original feature distribution relationship. We then utilize the similarity matrix to perform feature propagation (i.e., aggregation) of *K* steps. As the number of propagation steps increases, the scope of feature propagation centered on each node gradually expands, thereby capturing more global information for each node. Finally, an element-wise mean-pooling operation is applied to aggregate the output of feature propagation of different steps. This aggregation of multi-step (i.e., multi-scale) feature propagation can improve the model representation capability. We conducted extensive experiments on a variety of public datasets and show the competitive performance of our method compared to various popular architectures.

The rest of this article is organized as follows. Section 2 reviews the related work. In Section 3, we describe our proposed method, and then perform an experimental evaluation in Section 4. Finally, in Section 5, we summarize our contributions and future work.

## 2. Related Work

Given a graph G=(V,E), where V and E are the set of n nodes and the set of edges respectively. Let $X_{i}$ nodes and the set of edges respectively. Let denote the feature vector for node i and $Y_{i}$ denote the true label. All node features (labeled and unlabeled) are represented by $X=[X_{1},X_{2},\ldots ,X_{n}{]}^{T}\in {\mathbb{R}}^{n\times c}$, with a *c*-dimensional feature vector for per node. Let L denote the set of labeled nodes and $Y\in {\mathbb{R}}^{|L|\;\times f}$ denote the one-hot label matrix, where f is the number of classes.

### 2.1. Graph-Based Semi-Supervised Learning

Generally, graph-based semi-supervised learning can be defined by the following loss function:

(1) $$ℒ={ℒ}_{\mathrm{label}}+\lambda {ℒ}_{reg}$$

${ℒ}_{\mathrm{label}}$ and ${ℒ}_{reg}$ are defined as,

(2) $${ℒ}_{\mathrm{label}}=\sum_{i\in L}l\left(f\left(X_{i}\right),Y_{i}\right)\;\mathrm{and}{ℒ}_{\mathrm{reg}}=\sum_{(i,j)\in E}A_{ij}{\left\|f\left(X_{i}\right)-f\left(X_{j}\right)\right\|}^{2}$$

where ${ℒ}_{\mathrm{label}}$ is the standard supervised loss for loss function l and ${ℒ}_{reg}$ is called as graph Laplacian regularization. ${ℒ}_{reg}$ can ensure that connected nodes have a similar model output, λ∈R is the regularization coefficient. $f(X_{i})$ denotes the label prediction of node i, and $f(X_{i})$ is predicted by learning both labeled and unlabeled nodes simultaneously. A represents an adjacency matrix or other graph construction and $A_{ij}$ denotes a certain relationship between graph nodes i and j.

Graph-based semi-supervised learning has been a popular research field in the past few years. By using the graph structure to aggregate the feature information from the labeled and unlabeled nodes, learning can be done with very few labels. There are already many methods for graph-based semi-supervised learning. The label propagation algorithm [21] uses labeled node label information to predict unlabeled node label information and uses the relationship between samples to establish a complete graph model. ManiReg [22] calculates the supervised loss on the labeled nodes and calculates the unsupervised loss on all nodes using the graph Laplacian regularization. SemiEmb [23] regularizes a deep neural network with an embedding-based regularizer. Planetoid [7] is a method based on sampling, and the authors derived a sampling algorithm based on random walks to obtain the positive and negative contexts for each data point.

### 2.2. Graph Neural Networks

Graph neural networks (GNNs) are an extension of neural networks to structured data encoded as graphs, which update the features $X_{i}^{\left(t-1\right)}$ of node i∈V of node in layer t−1 by aggregating local information via

(3) $$X_{i}^{\left(t\right)}=f_{\Theta }^{\left(t\right)}\left(X_{i}^{\left(t-1\right)},{\left\{X_{w}^{\left(t-1\right)}\right\}}_{w\in \;N(i\;)}\right)\;,\;i.e.,\;X_{i}^{\left(t\right)}=\sigma \;\left(\Theta^{\left(t\right)}\sum_{w\in \;N(i)\cup \{i\}}C_{i,\;w}^{\left(t-1\right)}X_{i}^{\left(t-1\right)}\right)$$

where N(i) is the set of neighbors of node i in the graph and $f_{\Theta }^{\left(t\right)}$ is a differential function parameterized by weights $\Theta^{\left(t\right)}$. In some current implementations, $C_{i,w}^{\left(t-1\right)}$ is defined as either static [24], structure- [8] or data-dependent [25]. 

GNNs were originally introduced as extensions of recurrent neural networks. They learn a target node’s representation by propagating neighbor information in an iterative manner until a stable fixed point is reached. However, as the weights are shared among all nodes, GNNs can also be interpreted as extensions of convolutional neural networks on a 2D grid to general graphs and aim at addressing graph-related tasks in an end-to-end manner. GNNs have been successfully applied in various applications, such as community detection [26,27], molecular activation prediction [28], matrix completion [29], combinatorigal optimization [30], and detecting similar binary codes [31].

### 2.3. Semi-Supervised Learning with GCN

The GCN [14] model is a special case of GNNs, and it is a simple but powerful architecture that stacks two layers of specific propagation and perceptron. Given the input feature matrix X and adjacency matrix A, the output of the two-layer GCN model can be defined as:

(4) $$Z=softmax(\hat{A}\mathrm{ReLU}(\hat{A}X\Theta^{\left(1\right)})\Theta^{\left(2\right)})$$

Here, $\hat{A}={\tilde{D}}^{-1/2}\tilde{A}{\tilde{D}}^{-1/2}$ is a symmetric normalized adjacency matrix and $\tilde{A}=A+I$, where $I\in {\mathbb{R}}^{n\times n}$ is the identity matrix and $\tilde{D}$ is a diagonal degree matrix with ${\tilde{D}}_{ii}=\sum_{j}{\tilde{A}}_{ij}$. ReLU is a rectified linear activation function where ReLU(x)=max {0,x}, and $softmax(x_{i})=\frac{1}{m}exp(x_{i})$ with $m=\sum_{i}exp(x_{i})$ is applied row-wise. The weight matrices $\Theta^{\left(1\right)}\in {\mathbb{R}}^{c\times h}$ and $\Theta^{\left(2\right)}\in {\mathbb{R}}^{h\times f}$ are trained to minimize the cross-entropy loss over all labeled examples L:

(5) $${ℒ}_{\mathrm{GCN}}=-\sum_{i\in L}\sum_{l=1}^{f}Y_{il}\ln Z_{il}$$

The GCN model combines graph structure and node features in the convolution, where the features of unlabeled nodes are mixed with those of nearby labeled nodes. As the GCN model leverages the features of unlabeled nodes in training, it only requires fewer labeled nodes to achieve better prediction results.

## 3. The Proposed Method

In this section, we introduce our method in two steps. First, we introduce the process of calculating the similarity matrix. Then, we introduce the multi-scale aggregation graph neural network method based on the similarity matrix.

Compared with the previous graph convolution models, our method has two innovations: (i) We no longer use the adjacency matrix to participate in node feature update. We construct a similarity matrix to take the place of the adjacency matrix, which can distinguish the relative importance of neighbor nodes for the target node in the feature update; (ii) We use an average encoding with skip connections in the feature propagation of each layer, which is an aggregation of multi-scale feature propagation. Compared to previous single-scale feature propagation methods (e.g., GCN [8] and SGC [15]), this multi-scale aggregation can not only retain adequate lower-order neighbors’ information, but also obtain more global information.

**Figure 1:**
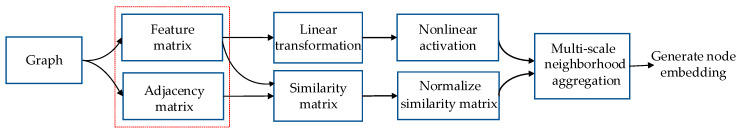
Flow illustration of the proposed architecture.

The flow illustration of the proposed method is shown in Figure 1. This similarity matrix needs to be obtained in advance. During training, the obtained similarity matrix can be used directly. First, we need to calculate the similarity matrix and normalize the similarity matrix. Since the adjacency matrix can be easily obtained, we can obtain the similarity matrix according to the adjacency matrix and the original feature matrix. Then, we need to build network architecture of the proposed method, mainly divided into three steps: (i) we need to input the feature matrix into a fully connected network for linear transformation to reduce the feature dimensions; (ii) nonlinear activation is performed to obtain node hidden representations; (iii) multi-scale neighborhood aggregation is performed to generate node embeddings. A multi-layer network architecture can be generated by repeating these three steps.

### 3.1. Calculating Similarity Matrix

Following the notation in Section 2, $X=[X_{1},X_{2},\ldots ,X_{n}{]}^{T}\in {\mathbb{R}}^{n\times c}$ is the feature matrix, composed of the features of all labeled and unlabeled nodes, where $X_{i}\in {\mathbb{R}}^{c}$ is the c-dimensional feature vector of node i and n is the number of all nodes. The graph structure is represented by the adjacency matrix $A\in {\mathbb{R}}^{n\times n}$. Generally, the feature similarity between two nodes is compared by calculating their feature distance. The smaller the feature distance is, the greater the similarity, and conversely, the smaller the similarity. In our model, we use the Manhattan distance to calculate the feature similarity between two nodes.

Nodes i and j are adjacent nodes in the graph, and the Manhattan distance between their features can be gained by the following formula:

(6) $$d_{ij}=|X_{i1}-X_{j1}\left|+\right|X_{i2}-X_{j2}|+\cdots +|X_{ic}-X_{jc}|=\sum_{\varepsilon =1}^{c}\left|X_{i\varepsilon }-X_{j\varepsilon }\right|$$

Calculating the similarity coefficient between nodes i and j via

(7) $$\alpha_{ij}=\frac{1}{\mu +\exp(d_{ij})}$$

where μ is the smoothing parameter. By Equation (7), a smaller feature distance will obtain a larger similarity coefficient.

The similarity matrix $S\in {\mathbb{R}}^{n\times n}$ is defined by

(8) $$S_{ij}=\left\{ \begin{array}{ll} \alpha_{ij}, & (i,j)\;\in E\\ \frac{1}{\mu +1}, & i=j\\ 0, & \mathrm{otherwise} \end{array}\right.$$

where (i,j)∈E means that nodes i and j are adjacent nodes in the graph, similarity coefficient $\alpha_{ij}$ of adjacent nodes i and j is calculated by Equations (6) and (7). 

Algorithm 1 describes the process of calculating the similarity matrix S. Note that the input feature matrix needs to be normalized before calculating the similarity matrix. Otherwise, if the similarity gap of different neighboring nodes is too great, it will lead to lower classification accuracy. Actually, S can be regarded as an adjacency matrix with weights. S is used in feature propagation, which can distinguish the relative importance of neighbors based on the similarity of original features between the target node and neighbors. These neighbors with higher similarity tend to play a more important role in feature propagation.

**Algorithm 1.** Calcul ate Similarity Matrix S1: Input: feature matrix $X\in {\mathbb{R}}^{n\times c}$, adjacency matrix $A\in {\mathbb{R}}^{n\times n}$2: output: similarity matrix $S\in {\mathbb{R}}^{n\times n}$3: Perform normalization $X\leftarrow {\hat{D}}^{-1}X$ with diagonal matrix ${\hat{D}}_{ii}=\sum_{\varepsilon =1}^{c}X_{i\varepsilon }$4: Initialize S with zeros 5: for i=0 to n
do 6:  N(i)= Non-zero($A_{i}$) // N(i) is the set of 1-hop neighbors of node i7:  for j in N(i)∪{i} do8:    $d_{ij}=\sum_{\varepsilon =1}^{c}\left|X_{i\varepsilon }-X_{j\varepsilon }\right|$ // calculating the feature distance of nodes i and j9:     $S_{ij}={\left(\mu +\exp(d_{ij})\right)}^{-1}$ // calculating the feature similarity of nodes i and j10:  end for 11: end for 12: return S

**Figure 2:**
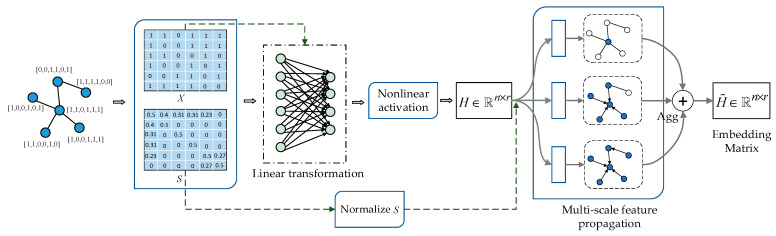
Diagrammatic representation of the proposed architecture.

We need to use the similarity matrix (calculated in Algorithm 1) for the proposed architecture to perform multi-scale feature propagation, specific as shown in Figure 2, where X represents the feature matrix and S represents the feature matrix and represents the similarity matrix. First, we need to perform linear transformation and nonlinear activation on the feature matrix X to obtain the hidden feature representation $H\in {\mathbb{R}}^{n\times r}$, where n represents the number of nodes in the graph and r represents the hidden feature dimensions. Next we use the normalized similarity matrix to perform multi-scale feature propagation on hidden feature representation H. Then we use an aggregator to aggregate the output of multi-scale feature propagation to generate an embedding matrix $\tilde{H}\in {\mathbb{R}}^{n\times r}$. In Figure 2, “+” means aggregator, here we use mean-pooling as aggregator. For the proposed method, we will describe it in further detail in Section 3.2. 

### 3.2. MAGN Model

In the GCN, hidden representations of each layer are aggregated among neighbors that are one hop away. This implies that after *K* layers, a node extracts feature information from all nodes that are *K* hops away in the graph. Each GCN layer has only a size-1 feature extractor, so more layers are needed to obtain adequate global information. Different from GCN, we explore a set of size-1 up to size-*K* feature extractors in each layer to extract multi-scale neighborhood features for node representations. Considering that if only a size-*K* feature extractor is used, the resulting model is linear; this linear approximation leads to information loss and classification accuracy degradation. For example, the SGC [15] model only uses a fixed size-*K* feature extractor. Although the training time of the SGC model is reduced to a record low, its performance on some benchmark datasets is degraded compared with GCN. In contrast, using a set of size-1 up to size-*K* feature extractors (e.g., in our MAGN) can avoid the linear approximation and increase the representation ability. More importantly, our model needs fewer layers to obtain adequate global information.

We first normalize the similarity matrix S, and let $\tilde{S}$ denote the “normalized” similarity matrix:

(9) $$\tilde{S}=D^{-1}S$$

where D is a diagonal matrix and $D=\mathrm{diag}(\sum_{j=1}^{n}S_{1j},\ldots ,\sum_{j=1}^{n}S_{nj})$. For the overall model, we consider a multi-layer MAGN with the following layer-wise propagation rule:

(10) $${\tilde{H}}^{\left(t\right)}=\frac{1}{K+1}\sum_{k=0}^{K}\left({\tilde{S}}^{k}\sigma ({\tilde{H}}^{\left(t-1\right)}\Theta^{\left(t\right)}\right))$$

where ${\tilde{H}}^{\left(t-1\right)}$ is the feature representation of the (t−1)-th layer; ${\tilde{H}}^{\left(0\right)}$ equals to the input feature matrix X. $\Theta^{\left(t\right)}$ is a layer-specific trainable weight matrix, and σ denotes ReLU activation function. Note that the input feature matrix X consists of all node features (i.e. labeled and unlabeled), and we can utilize the similarity matrix to combine feature information from labeled and unlabeled nodes to generate node embedding. ${\tilde{S}}^{K}$ represents the *k*-th power of $\tilde{S}$ and we define ${\tilde{S}}^{0}$ as the identity matrix; ${\tilde{S}}^{1}$ to ${\tilde{S}}^{K}$ represent a set of size-1 up to size-*K* feature extractors, which are used to extract multi-scale neighborhood features. When k>1, calculating the *k*-th power of $\tilde{S}$ can transfer the similarity from 1-hop neighbors to *k*-hop neighbors, which is equivalent to adding an edge directly connected to the *k*-hop neighbors for each node. Therefore, our model can directly obtain feature information from *k*-hop neighbors for each node by learning the *k*-th power of $\tilde{S}$. With the increase in *k*, the scope of feature extraction (i.e., feature propagation) gradually expands, which can capture more global information.

In each MAGN layer, feature representations are updated in four stages: linear transformation (i.e., feature learning), nonlinear activation, feature propagation, and multi-scale aggregation. We adopt a strategy of learning first and then propagating, using a trainable weight matrix to perform linear transformation to degrade the feature dimensions, and then perform multi-scale feature propagation on low-dimensional features. Compared with the strategy of propagating first and then learning, using this method can reduce computational complexity and shorten the training time. We describe each step in detail.

**Linear transformation and nonlinear activation**. Each MAGN layer first performs linear transformation by a trainable weight matrix $\Theta^{\left(t\right)}$ to learn node features. Then, a nonlinear activation function ReLU is applied pointwise before outputting hidden representation $H^{\left(t\right)}$:

(11) $$H^{\left(t\right)}=\mathrm{ReLU}({\tilde{H}}^{\left(t-1\right)}\Theta^{\left(t\right)})$$

In particular, ${\tilde{H}}^{\left(0\right)}=X$ and $H^{\left(1\right)}=\mathrm{ReLU}(X\;\Theta^{\left(1\right)})$ when t=1.

**Feature propagation and multi-scale aggregation**. After the feature transformation, we use the “normalized” similarity matrix $\tilde{S}$ to generate a set of size-1 up to size-*K* feature extractors for multi-scale feature propagation. Then, a mean-pooling operation is applied to aggregate hidden representation $H^{\left(t\right)}$ and the output of multi-scale feature propagation. In summary, the final feature representation updating rule of the *t*-th layer is:

(12) $${\tilde{H}}^{\left(t\right)}=meanpool\;(H^{\left(t\right)}+{\tilde{S}}^{1}H^{\left(t\right)}+\cdots +{\tilde{S}}^{K}H^{\left(t\right)})=\frac{1}{K+1}\sum_{k=0}^{K}\left({\tilde{S}}^{k}H^{\left(t\right)}\right)$$

where ${\tilde{S}}^{1}H^{\left(t\right)}$ to ${\tilde{S}}^{K}H^{\left(t\right)}$ denote feature propagation on different scales of the graph and can directly obtain feature information across near or distant neighbors. ${\tilde{S}}^{0}H^{\left(t\right)}=H^{\left(t\right)}$ is added to keep more of its own feature information for each node. ${\left({\tilde{S}}^{k}\right)}_{ij}$ represents the probability of starting at node i to complete k steps of the random walk and finally reaching node j. The *k*-th power of $\tilde{S}$ contains statistics from the *k*-th step of a random walk on the graph. Therefore, ${\tilde{S}}^{1}$ to ${\tilde{S}}^{K}$ can combine information from different step-sizes (i.e., graph scales). The output row-vector of individual node i is:

(13) $${\tilde{H}}_{i}^{\left(t\right)}=\frac{1}{K+1}\;(\sum_{k=0}^{K}\sum_{j\in \;N_{k}(i)\cup \{i\}}{\left({\tilde{S}}^{k}\right)}_{ij}H_{j}^{\left(t\right)})$$

where $N_{k}(i)$ is an empty set if k=0; otherwise, it is the set of *k*-hops neighbors of node i. For an individual node, its final feature representation in the *t*-th layer is the aggregation of multi-hops neighbors’ features and its own features.

It is worth noting that the propagation scheme of this model does not require any additional parameters (i.e., trainable weights) to train, in contrast to models such as GCN, which usually require more parameters for each additional propagation function. Therefore, each layer of this model can propagate farther with very few parameters.

**Prediction function**. The output layer is similar to GCN, and we use a softmax function to predict the labels. The class prediction $\hat{Z}$ of a *t*-layer MAGN can be written as:

(14) $$\hat{Z}=softmax({\tilde{H}}^{\left(t\right)})$$

**Loss function**. The loss function is defined as the cross-entropy of prediction over the labeled nodes:

(15) $${ℒ}_{MAGN}=-\sum_{i\in L}\sum_{l=1}^{f}{\hat{Y}}_{il}\ln{\hat{Z}}_{il}$$

where L is the set of labeled nodes used as the training set and f is the number of classes. $\hat{Y}\in R^{|\;L|\times f}$ represents the corresponding true label matrix for the training set, and ${\hat{Y}}_{il}$ is 1 if the node i belongs to class l; otherwise, it is 0. ${\hat{Z}}_{il}$ is the predicted probability that node i is of class l.

**Figure 3:**
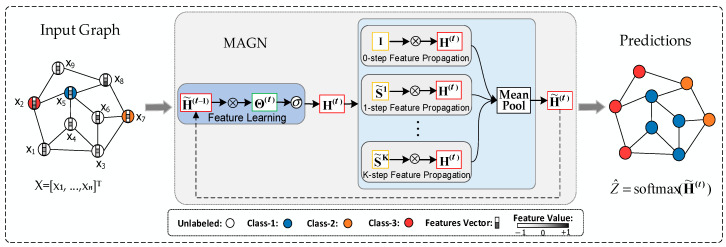
Schematic depiction of the MAGN network for semi-supervised learning.

Our model can learn based on the features of both labeled and unlabeled nodes simultaneously, and only use the training set labels to calculate the loss (i.e., only the training set labels are used for learning). Therefore, our model is a semi-supervised learning method for graphs. The proposed MAGN model for semi-supervised learning is schematically depicted in Figure 3, on the left is an input graph, in the middle is a *t*-layer MAGN model, and on the right is an output graph, where $\tilde{S}$ is the normalized similarity matrix, I is the identity matrix and equal ${\tilde{S}}^{0}$. × is the matrix-matrix multiply operator, and σ is the ReLU activation function. ${\tilde{H}}^{\left(t-1\right)}$ is the input feature representation, ${\tilde{H}}^{\left(0\right)}=X$, and ${\tilde{H}}^{\left(t\right)}$ is the output feature representation. Overall, Figure 3 shows that labeled and unlabeled nodes are used to predict the labels of unlabeled nodes via the MAGN model.

## 4. Experiments

In this section, we test our proposed MAGN model on semi-supervised node classification tasks. We first introduce the four datasets used in the experiments. Then, we list the compared methods and some implementation details. Finally, we test the classification accuracy of our model on fixed data splits and random data splits and compare it with some popular methods.

### 4.1. Datasets

For our experiments, we used three well-known citation network datasets: Cora and CiteSeer from [32], and PubMed from [33]. In the three citation network datasets, nodes represent documents and edges are citation links. We also introduce a co-author dataset for the node classification task: Coauthor CS (from [34]), which is a co-authorship graph. Here, nodes are authors, that are connected by an edge if they co-authored a paper, and the class labels indicate the most active research field of each author. All datasets use a bag-of-words representation of the papers’ abstracts as features. These four datasets can be downloaded from https://github.com/shchur/gnn-benchmark/tree/master/data (accessed on 5 April 2020). The details of these four datasets are summarized in Table 1.

### 4.2. Compared Methods

We compare our methods with other methods, including feature-based multi-layer perceptron (MLP), manifold regularization (ManiReg [22]), semi-supervised embedding (SemiEmb [23]), label propagation (LP [21]), and Planetoid [7], adaptive receptive paths (GeniePath [35]), mixture model networks (MoNet [13]), graph convolutional networks (GCN [8]), simplifying graph convolutional networks (SGC [15]), deep graph infomax (DGI [36]), graph attention networks (GAT [25]), and scalable inception graph neural networks (SIGN [37]). Among them, MLP is a kind of multi-layer fully connected network; ManiReg, SemiEmb, LP, and Planetoid do not belong to graph neural network algorithms but are traditional graph-based semi-supervised learning methods; GCN, GeniePath, MoNet, SGC, DGI, GAT, and SIGN are popular graph neural network algorithms.

### 4.3. Implementation

In practice, we make use of Pytorch for implementation by using sparse–dense matrix multiplications. In the concrete implementation, the similarity matrix is a sparse matrix, and the input feature matrix and the learnable weight matrices are dense matrices. All the experiments were conducted on a computer with an Nvidia GeForce RTX 2080 Ti GPU (11 GB GPU memory, Dell, Beijing and China). For the experimental parameter settings, as shown in Table 2 and Table 3, where dropout rate [38], $L_{2}$ regularization, and early stopping are added to avoid overfitting, all the experimental methods used Adam optimizer [39]. In addition, for the parameter settings for random data splits, we set different *K* values for our method in different datasets. For Cora, *K* = 6. For CiteSeer and PubMed, *K* = 5. For Coauthor CS, *K* = 4.

### 4.4. Result

#### 4.4.1. Fixed Data Splits

In this first experiment, we use the fixed data splits from [7], as they are the open standard data splits in the literature. Fixed split is the most commonly used data splitting method. Many works use it as a standard split to test the classification performance of their methods. The fixed split has only one split; according to [7], all experiments use 20 nodes per class as the training set. The size of the training set is determined by the number of classes, which can ensure that the labels of all types of nodes are used for training. According to the number of classes in Table 1, we can obtain the size of training sets for Cora, CiteSeer, and PubMed to be 140, 120, and 60, respectively. The number of training nodes in these three datasets accounts for a very small proportion of the total number of nodes, which are 5.2% (Cora), 3.6% (CiteSeer), and 0.3% (PubMed), respectively. There is no standard fixed split for Coauthor CS, and we use it in random data splits. Furthermore, the validation and test sets of these three datasets keep the same size, with 500 nodes for the validation and 1000 nodes for the test. We ran 20 different random initializations for our method and the parameter settings are provided in Table 2. The experimental results with a fixed split are reported in Table 4 in percentages. The classification accuracy of all the compared methods is collected from [8,13,15,25,35,36].

From Table 4, we can see that although our method performs lower than GAT on CiteSeer, it surpasses all compared methods on Cora and PubMed. It is worth noting that in Table 4, the classification accuracies of these graph neural network algorithms are much higher than that of MLP and traditional graph-based semi-supervised learning methods. MLP is a kind of multi-layer fully connected network and cannot use graph structure information for learning. Therefore, the classification accuracy is relatively low. These traditional graph-based semi-supervised learning methods only leverage graph structure information and known label information to train the semi-supervised classifier, but the feature information of nodes is ignored in training, which leads to a lower classification accuracy.

**Figure 4:**
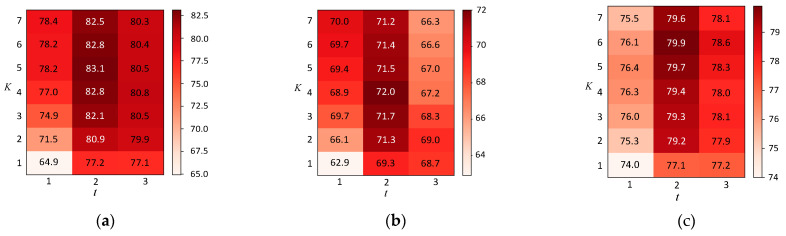
Average accuracy on different datasets when varying *K* and *t.* (**a**) MAGN on Cora. (**b**) MAGN on CiteSeer. (**c**) MAGN on PubMed.

For fixed data splits, we also report the classification accuracy of our method with different K and different t (t represents the number of network layers). Except for K and t, the other experimental settings are the same as in Table 4 and are provided in Table 2. Experimental results are shown in Figure 4 in percent. Here, we report the average accuracy of running 20 different random initializations for our models. From the figure, it can be found that the model performs better when t=2 and K∈[2, 7]. Although the model has only two layers, it can obtain adequate global information by adjusting the value of K.

**Figure 5:**
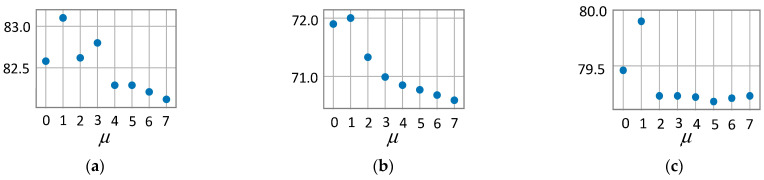
Average accuracy of MAGN for varying smoothing parameter μ. (**a**) MAGN on Cora. (**b**) MAGN on CiteSeer. (**c**) MAGN on PubMed.

In addition, the smoothing parameter μ also has a certain impact on model performance. To this end, we further test the model performance with different μ on fixed data splits. The experimental results are shown in Figure 5 as percentages; we can see that the model performs best when μ is set to 1. A larger μ does not improve the accuracy, which may be because a larger μ leads to the inability to better distinguish the relative importance of neighbor nodes. 

**Figure 6:**
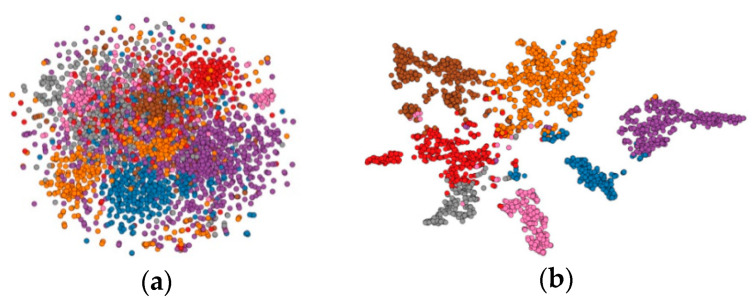
t-SNE visualization of the nodes from the Cora dataset. (**a**) Raw Cora dataset. (**b**) Trained Cora dataset.

Figure 6 shows the t-SNE [40] visualization of the nodes from the Cora dataset; the left one is t-SNE visualization of the nodes in the Cora dataset from the raw features and the right one from the Cora dataset is trained with a two-layer MAGN model using 5.2% of labels. Colors denote the node class; we can see that the features of different types of nodes can be well-distinguished after training.

#### 4.4.2. Random Data Splits

Since the fixed split has only one split, in order to better prove that our method has competitive performance, we use multiple random splits in this part. The training set allocated for each random split is different. In order to ensure the fairness of the following comparisons, we will set some same random seeds to ensure that our method and other compared methods have the same training, validation, and test sets in each random split.

Next, following the settings of Buchnik and Cohen [41], for Cora, CiteSeer, and PubMed, we conducted experiments keeping the same size in training, validation, and test sets as in Table 4, but now selecting those nodes uniformly at random. For Coauthor CS, similarly, we randomly selected 20 nodes for each class as the training set, and randomly selected 500 nodes for validation and 1000 nodes for testing. We used 10 random seeds for 10 splits on each dataset, and every model was run with five different random initializations on each split, leading to a total of 50 runs per model. Note that all models have the same 10 random seeds, which can guarantee that all models have the same training, validation, and test sets for each split. Experimental results (i.e., average accuracy and standard deviation) with random data splits are shown in Table 5 and all experiments are completed by us. For every model, we selected the experimental settings that achieved the best accuracy, and these experimental settings are provided in Table 3.

From Table 5, we can see that our method achieves the best accuracy on all datasets. MLP has the lowest classification accuracy on all datasets. This is mainly because MLP does not make use of graph structure information in learning. Therefore, it cannot aggregate neighborhood information to generate node representations, which leads to poor performance in processing graph node classification tasks. The other compared methods are basically shallow single-scale aggregation methods, and the feature information obtained does not exceed the 2-hop neighborhood. Therefore, these models have difficulty obtaining adequate global information. Our model is based on multi-scale neighborhood aggregation, and only a small number of layers is needed to obtain adequate global information, which leads to improved classification accuracy. In Table 6, we further compare the number of parameters (i.e., trainable weights) that need to be trained on each dataset for these different methods.

In Table 6, we can see that, except for SGC, our method’s number of parameters are on par or lower than other compared methods. The main reason is that the propagation scheme of our method does not require any additional parameters to train, which results in a relatively small total number of parameters. SGC is usually a single-layer graph convolution method, and two or more layers will cause model performance degradation. Therefore, SGC can be trained with few parameters.

**Figure 7:**
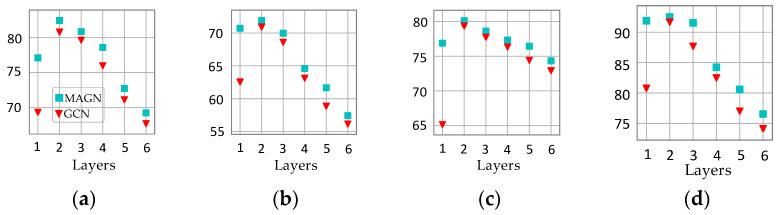
Average accuracy of MAGN and GCN for varying numbers of network layers. (**a**) Cora. (**b**) CiteSeer. (**c**) PubMed. (**d**) Coauthor CS.

We also tested the performance of MAGN and GCN models with different network layers on random data splits; the experimental results are shown in Figure 7. From the figure, we can see that both MAGN and GCN achieve the highest accuracy when the number of network layers is 2. Deep neural networks do not improve the accuracy, which may be due to the simple bag-of-words features and the small training set size. However, it is worth noting that the accuracy of the proposed MAGN model significantly outperforms GCN, especially when the number of network layers is 1.

#### 4.4.3. Random Splits with Different Training Set Sizes

Semi-supervised learning aims to obtain better learning results with less training data, which can greatly reduce the cost of manual labeling. In the above experiment evaluation, we selected 20 labeled nodes per class as the training set. In order to prove that our method still has better classification accuracy on less training data, we used some smaller training sets (that is, we selected fewer nodes per class as the training set).

Next, we randomly selected 5, 10, and 15 nodes per class, respectively, as the training set and kept the same size in the validation and test sets as in Table 5. Compared with Table 4 and Table 5, fewer labeled nodes were used for training. We used the same 10 random seeds as in Table 5 for determining the splits, and each model was run with five different random initializations on each split. The experimental settings of all models are the same as in Table 5 and are provided in Table 3. The experimental results are shown in Table 7, Table 8, Table 9 and Table 10.

It can be seen in Table 7, Table 8, Table 9 and Table 10 that the average accuracy of the proposed MAGN model outperforms the compared models on all datasets, and the model performance is relatively stable. This demonstrates that our model still maintains a better classification accuracy with fewer labeled nodes.

## 5. Conclusions

In this paper, we proposed a novel method for semi-supervised learning on graph-structured data. We first constructed a similarity matrix based on the original feature distribution of adjacent node pairs. Then, we used the similarity matrix to generate a set of feature extractors to extract multi-scale neighborhood features. Compared with traditional graph convolution methods, our method can distinguish more than the relative importance of neighbor nodes in feature propagation, and more importantly, it only requires a small number of layers to obtain sufficient global information. In addition, our method can aggregate feature information from unlabeled nodes by encoding graph structure and node features, which is conducive to semi-supervised learning. Extensive experiments demonstrate that our method outperforms other state-of-the-art methods in the case that labeled data is extremely scarce. For future work, we plan to extend our methods to address other (larger) graph datasets.

## Figures and Tables

**Table 1:** The Statistics of Datasets.

Dataset	Type	Classes	Features	Nodes	Edges
Cora	Citation	7	1433	2708	5429
CiteSeer	Citation	6	3703	3327	4732
PubMed	Citation	3	500	19,717	44,338
Coauthor CS	Co-author	15	6805	18,333	81,894

**Table 2:** Experimental settings for fixed data splits.

	Setting	Hidden Sizes	Learning Rate	Dropout Rate	$L_{2}\;\mathrm{Reg}$	Epochs	Early Stopping	Layers	*K*
Dataset									
Cora		64	0.002	0.5	0.0005	250	10	2	5
PubMed		64	0.01	0.5	0.0005	100	10	2	6
CiteSeer		64	0.01	0.5	0.0005	150	10	2	4

**Table 3:** Experimental settings for random data splits.

	Setting	Hidden Sizes	Learning Rate	Dropout Rate	$L_{2}\;\mathrm{Reg}$	Epochs	Early Stopping	Layers
Method								
MLP		64	0.01	0.5	0.0005	200	10	2
Geniepath		64	0.005	0.4	0.0005	10,000	200	2
SGC		--	0.2	0.5	0.000005	200	10	1
GCN		64	0.01	0.5	0.0005	200	10	2
SIGN		128	0.0005	0.5	--	200	10	5
DGI		512	0.001	--	--	5000	20	2
GAT		64	0.01	0.6	0.0005	10,000	100	2
MAGN		64	0.005	0.5	0.0005	200	10	2

**Table 4:** Classification accuracy with a fixed split of data. The highest accuracy in each column is highlighted in bold.

	Dataset	Cora	CiteSeer	PubMed
Method				
MLP		55.1	46.5	71.4
ManiReg		59.5	60.1	70.7
SemiEmb		59.0	59.6	71.1
LP		68.0	45.3	63.0
Planetoid		75.7	64.7	77.2
GCN		81.5	70.3	79.0
GeniePath		--	--	78.5
MoNet		81.7 ± 0.5	--	78.8 ± 0.3
SGC		81.0 ± 0.0	71.9 ± 0.1	78.9 ± 0.0
DGI		82.3 ± 0.6	71.8 ± 0.7	76.8 ± 0.6
GAT		83.0 ± 0.7	**72.5 ± 0.7**	79.0 ± 0.3
MAGN		**83.1 ± 0.3**	72.0 ± 0.2	**79.9 ± 0.1**

**Table 5:** Classification accuracy with random split of the data. The highest accuracy in each column is highlighted in bold.

	Dataset	Cora	CiteSeer	PubMed	Coauthor CS
Method					
MLP		59.23 ± 1.09	57.87 ± 1.61	58.94 ± 1.12	88.11 ± 0.76
GCN		80.77 ± 1.14	70.89 ± 1.22	79.33 ± 1.34	91.68 ± 0.64
GeniePath		79.04 ± 1.46	70.34 ± 1.16	78.52 ± 1.61	91.01 ± 0.97
SGC		80.32 ± 1.17	70.48 ± 0.94	78.39 ± 1.35	90.93 ± 0.96
SIGN		80.85 ± 1.65	68.13 ± 1.52	79.57 ± 1.94	92.02 ± 0.86
DGI		81.84 ± 1.21	71.55 ± 0.62	78.25 ± 1.53	91.09 ± 0.85
GAT		81.19 ± 1.27	71.01 ± 0.72	79.42 ± 1.21	91.33 ± 0.74
MAGN		**82.44 ± 1.05**	**71.89 ± 0.74**	**80.11 ± 1.03**	**92.53 ± 0.45**

**Table 6:** Number of parameters for different methods.

	Dataset	Cora	CiteSeer	PubMed	Coauthor CS
Method					
MLP		92,160	237,376	32,192	436,480
GeniePath		149,632	294,784	89,664	493,952
SGC		8598	22,218	1500	102,075
GCN		92,160	237,376	32,192	436,480
SIGN		1,297,920	3,041,280	580,992	5,424,768
DGI		999,424	2,161,152	194,304	1,811,456
GAT		92,302	237,516	32,326	436,638
MAGN		92,160	237,376	32,192	436,480

**Table 7:** Classification accuracy for different training set sizes on Cora. The highest accuracy in each column is highlighted in bold.

	Size	5 Per Class	10 Per Class	15 Per Class
Method				
MLP		38.35 ± 3.88	55.04 ± 2.74	55.99 ± 1.07
Geniepath		64.20 ± 3.89	73.30 ± 2.44	77.43 ± 1.34
SGC		66.28 ± 3.66	75.45 ± 2.37	78.65 ± 1.38
GCN		66.87 ± 3.89	75.43 ± 2.23	78.73 ± 1.43
SIGN		64.53 ± 4.81	74.96 ± 2.65	78.18 ± 1.61
DGI		71.01 ± 2.82	77.65 ± 2.08	79.77 ± 1.38
GAT		68.12 ± 3.91	77.51 ± 2.16	79.68 ± 1.11
MAGN		**71.64 ± 3.32**	**78.16 ± 1.80**	**80.38 ± 1.53**

**Table 8:** Classification accuracy for different training set sizes on CiteSeer. The highest accuracy in each column is highlighted in bold.

	Size	5 Per Class	10 Per Class	15 Per Class
Method				
MLP		41.79 ± 5.29	51.11 ± 2.78	54.29 ± 2.28
Geniepath		56.99 ± 4.13	64.47 ± 2.27	67.98 ± 1.32
SGC		56.01 ± 6.36	64.44 ± 2.43	67.49 ± 1.41
GCN		58.31 ± 5.41	65.97 ± 2.28	68.54 ± 1.47
SIGN		52.67 ± 5.05	61.19 ± 2.45	64.84 ± 1.69
DGI		59.68 ± 4.82	66.09 ± 2.11	69.13 ± 1.51
GAT		59.19 ± 5.67	65.81 ± 2.23	68.38 ± 1.31
MAGN		**60.14 ± 5.15**	**66.48 ± 1.75**	**69.47 ± 1.27**

**Table 9:** Classification accuracy for different training set sizes on PubMed. The highest accuracy in each column is highlighted in bold.

	Size	5 Per Class	10 Per Class	15 Per Class
Method				
MLP		37.78 ± 3.86	49.30 ± 3.26	54.36 ± 1.94
Geniepath		67.43 ± 5.52	73.84 ± 4.35	77.11 ± 2.43
SGC		67.95 ± 5.12	73.64 ± 4.12	76.89 ± 2.10
GCN		67.92 ± 5.19	74.44 ± 3.92	77.33 ± 2.72
SIGN		65.95 ± 5.77	74.35 ± 2.91	77.51 ± 1.69
DGI		66.35 ± 6.14	73.64 ± 4.01	76.72 ± 2.32
GAT		68.13 ± 5.32	74.52 ± 3.64	77.42 ± 2.14
MAGN		**70.39 ± 5.05**	**74.97 ± 3.53**	**77.96 ± 2.23**

**Table 10:** Classification accuracy for different training set sizes on Coauthor CS. The highest accuracy in each column is highlighted in bold.

	Size	5 Per Class	10 Per Class	15 Per Class
Method				
MLP		72.93 ± 2.90	82.92 ± 1.63	86.40 ± 1.23
Geniepath		88.45 ± 1.43	89.85 ± 1.32	90.72 ± 1.01
SGC		88.27 ± 1.51	89.89 ± 1.11	90.60 ± 1.24
GCN		88.44 ± 1.22	90.10 ± 1.16	91.17 ± 0.87
SIGN		88.34 ± 1.57	90.37 ± 1.14	91.34 ± 0.69
DGI		88.54 ± 1.12	90.45 ± 0.76	90.91 ± 0.74
GAT		88.52 ± 1.38	90.23 ± 1.05	91.05 ± 0.82
MAGN		**89.25 ± 1.10**	**91.03 ± 1.17**	**91.96 ± 0.71**

## Data Availability

All datasets of this study are publicly available at https://github.com/shchur/gnn-benchmark/tree/master/data (accessed on 23 March 2021).
